# Determinants of within-country variation in traffic accident mortality in Italy: a geographical analysis

**DOI:** 10.1186/1476-072X-6-49

**Published:** 2007-10-23

**Authors:** Giuseppe La Torre, Ed Van Beeck, Gianluigi Quaranta, Alice Mannocci, Walter Ricciardi

**Affiliations:** 1 Institute of Hygiene, Catholic University Rome, Italy; 2 Department of Public Health, Erasmus Medical Centre, Rotterdam, The Netherlands

## Abstract

**Objective:**

To identify determinants of regional differences in traffic accident mortality in Italy.

**Data and methods:**

Multiple linear regression models were conducted assessing the associations between regional differences in traffic mortality, case fatality and accident rates (dependent variables) with socio-demographic factors, and variables describing road behaviour, vehicles, infrastructure and medical care (independent variables). Data were derived from the National Institute of Statistics, the National Institute of Health and the Italian Automobile Club. In addition to analyses for the whole country of Italy, separate models were conducted for Northern and southern regions.

**Results:**

In Italy large regional differences in traffic mortality rates can be observed, ranging from 5.5 to 20 per 100.000 person-years. There is a North-South gradient with higher mortality rates in the Northern part of Italy. Strong predictors of regional differences in both traffic mortality and accident rates are the employment rate (directly associated) and alcohol use (directly associated). This is observed in the whole of Italy, and separately in Northern and southern regions.

**Conclusion:**

Our study has shown the need for regional policies to improve road behaviour to reduce traffic accident and mortality rates in identified high-risk areas.

## Introduction

It is estimated that in the European Union 1,3 million road traffic accidents occur each year, resulting in 1,7 million injuries and over 40 000 deaths annually. The direct and indirect costs of these accidents sums up to160 billion Euro, equal to 2% of the gross national product of the European Union [1]. Furthermore, road traffic injuries are a leading cause of death and disability in children and young people [2-4] and are the main cause of death in those under 45 year of age in the European Union [1]. Some statistics reveal that 70% of those injured or killed in traffic crashes are aged between 10–39 [5].

As a result of preventative measures and improvements of trauma care, traffic accident mortality in the industrialized world is decreasing since the early 1970s. The most important reduction being observed in Europe, especially during the decade from 1970 to 1980 [6]. Despite these improvements, however, road traffic accidents still represents a significant public health problem in Europe with considerable inequalities among population groups, reflected by large cross-country variation and within-country variation.

Few studies are available in the scientific literature regarding the determinants of within-country variation in traffic accident mortality, however the ones that do exist show that in some countries large and important regional differences in mortality rates may be present [6,7]. In these countries, regional differences in traffic accident mortality seem to reflect the influence of socio-demographic factors: for example, an inverse relationship between death rates of motor vehicle occupants and per capita income has been reported [7,8], and occupational status seems another important determinant of driver injury risk [9].

In Italy, around 270 000 road traffic accidents occur annually, causing almost 330 000 injuries and 7 000 deaths [10], and these figures are likely to be conservative as underreporting is a well-known phenomenon in traffic accidents statistics [6]. In Italy, regional differences in traffic accident mortality and its underlying determinants have not been studied so far, although this country is characterized by large regional differences in socio-economic development.

The aim of this study is to verify if in Italy regional differences exist in traffic accident mortality and to identify their determinants. The possible relationship between traffic accident mortality and socio-demographic factors, and between traffic accident mortality and variables describing road behaviour, road vehicles, infrastructural road characteristics and medical care, will be analyzed in order to discover the strongest predictors of regional mortality differences. Knowledge on the relationship between traffic accident mortality at regional level and socio-demographic factors or infrastructure and safety variables could contribute to our understanding of motor vehicle-related deaths. This may support the prevention of such avoidable mortality and help reducing existing inequalities in road traffic deaths within Italy and other countries.

## Methods

### Traffic data sources

Data concerning traffic accidents, traffic deaths and injured people by region for the period 1999–2001 were available from "Statistics of crash accidents Year 2001" edited by the National Institute of Statistics [11]; the same data for the year 2002 were available from the "Statistic Year-Book 2004", also edited by the National Institute of Statistics [12]. Traffic mortality and accident rates were calculated as number of deaths for crash accidents per 100 000/resident population, and number of traffic accidents per 100 000/resident population, respectively, using the 2001 resident reference population from the XIV General Census (Source of the data: National Institute of Statistics) [13]. In order to allow comparisons between regions, the mortality rates were standardized (direct method) by age and sex. Furthermore, we calculated case-fatality rates (traffic accident deaths/injured persons) for the years 1999, 2000, 2001 and 2002, as index of traffic accident severity. The average rates were calculated by dividing the sum of the rates for the years 1999–2002 by 4, assuming that the population was constant in this period (2001 population used as reference).

### Socio-demographic data

In order to study the association between traffic accident mortality and socio-demographic factors, we collected the following socio-demographic data:

▪ the regional employment rate;

▪ the degree of regional urbanization;

▪ the average per capita income by region.

The employment rate was calculated as the percentage of employed people in the age 15–64 years within the population in the corresponding age class. The regional employment rates for the years 1999, 2000, 2001 and 2002 were available from "The regional indicators for the evaluation of the development politics" edited by the National Institute of Statistics [14]; the degree of regional urbanization was available as a percentage of the urban population by region for the years 2001 and 2002 from the "Health system and population's health", also edited by the National Institute of Statistics [15]. Average rates, used for the analysis, were calculated for both the regional employment rate and for the degree of regional urbanization.

As far as the average per capita income by region is concerned, we used the average data from the period 1995–2001 of the "per capita wealth" as reported by the "Osservasalute Report 2003" [16].

### Road behaviour

We used prevalence of alcohol use in the Italian Regions as a measure of exposure, derived from the 2005 annual report of the Italian Observatory of Health in the Regions, concerning data on 2002 (Osservasalute 2005) [17].

### Road vehicles and infrastructure

We collected data regarding circulating vehicles (absolute figures), which were used to measure traffic density, as a measure of exposure [18].

For characteristics describing road infrastructure we collected information about the availability of highways (absolute figures) in the years 2000, 2001 and 2002 by region, from the Statistic Year-Books 2002, 2003 and 2004 edited by the National Institute of Statistics [19-21]. The average length of the highways by region was calculated and used for the analysis.

### Medical care

Finally, we collected data for each region, regarding medical care potentially linked to traffic deaths, such as availability of Magnetic Resonance Imaging scans (MRI-scan) in public and accredited hospitals (number of scans per 100 000 regional inhabitants as an indicator of advances in health care). These data were collected from the "Health system and population's health" (edited by the National Institute of Statistics) [16], and were used in the analysis because of the previously reported relationship between motor vehicle crash mortality and the availability of advanced emergency and trauma care [6,7,22].

### Statistical analysis

Spearman's correlation coefficient was calculated to assess the association between the three outcome variables (mortality rate, accident rate and case fatality rate).

We conducted several analyses in order to identify the statistically significant determinants of regional differences in mortality, case fatality and accident rates respectively. The association between possible determinants and the outcome variables was analysed using multiple regression models.

In the first analysis, the influence of socio-demographic factors was investigated in order to show the possible "social aetiology" of the regional differences.

Three socio-demographic variables by region were selected for the models:

▪ degree of urbanization;

▪ employment rate;

▪ per capita income.

The second multivariate approach was performed in order to evaluate the influence of regional differences in road behaviour, vehicles, infrastructure and medical care on mortality, case-fatality and accident rates.

These multiple linear regression models were performed using as covariates four variables representing different types of determinants:

▪ prevalence of alcohol use (road behaviour)

▪ number of circulating vehicles (road vehicles);

▪ length of highways (road infrastructure) ;

▪ availability of Magnetic Resonance scans (trauma care).

The three socio-demographic variables and the four other factors were subjected to separate multiple regression analyses because they concern two different levels of explanation [6].

In addition to analyses covering the whole country, separate analysis were conducted considering Northern (Piemonte, Valle d'Aosta, Lombardia, Trentino Alto Adige, Veneto, Friuli Venezia Giulia, Liguria, Emilia Romagna, Toscana, Umbria, Marche and Lazio) and Southern (Abruzzo, Molise, Campania, Puglia, Basilicata, Calabria, Sicilia and Sardegna) regions. We conducted separate analyses for northern and southern regions respectively, since Italy knows a North-South distinction in socio-economic development and health outcomes, particularly showing differences for several indicators for southern regions in respect with the rest of the Country [23].

For the analyses SPSS software was used, version 12.0 for Windows.

The multivariate approach was performed using the "backward elimination" procedure; the goodness of fit of the different linear regression models performed was evaluated using the statistic R^2^; the statistical significance was set at *p *< 0.05.

## Results

**Figure 1:**
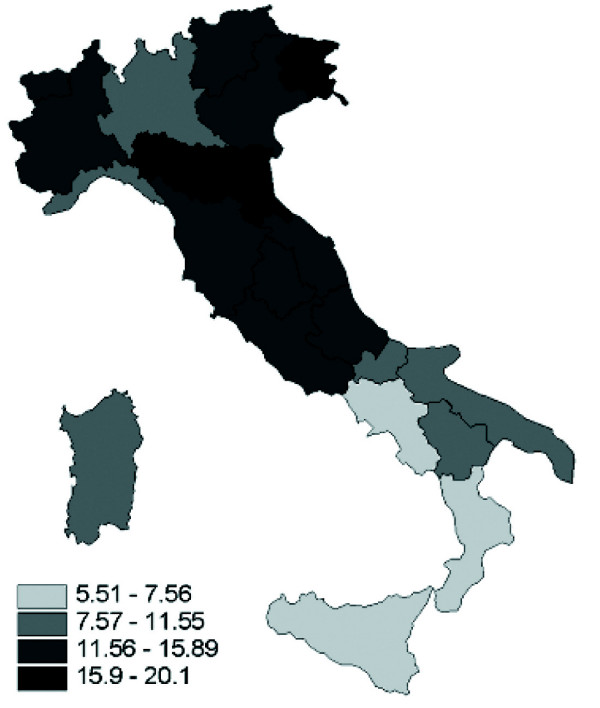
Traffic mortality rates per 100 000 person-years by region, Italy. 1999–2002.

As shown in Figure 1, in Italy regional differences in traffic mortality can be observed, ranging from 5.5 to 20 deaths per 100.000 person-years. Traffic mortality rates are higher in the northern regions, with a clear north-south decreasing gradient.

**Figure 2:**
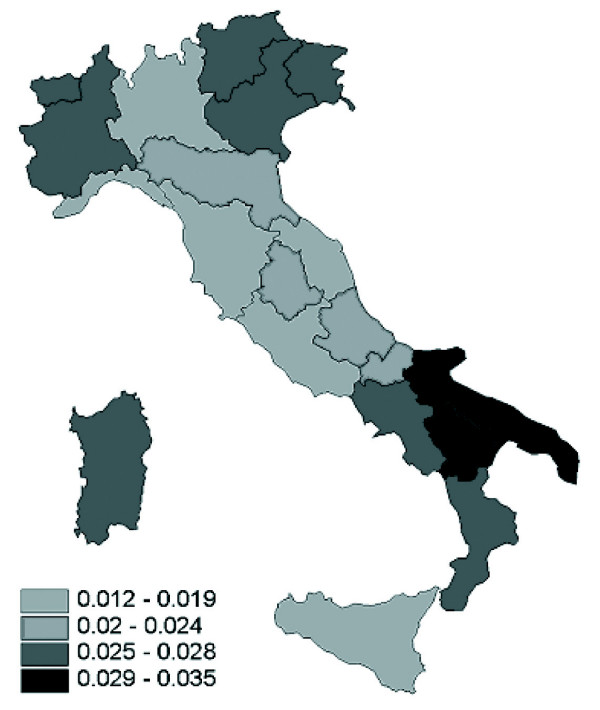
Case-fatality rates per 100.000 person-years by region, Italy,1999–2002.

Figure 2 shows the average case-fatality rates in the years 1999–2002; regional differences can also be observed for case-fatality rates. The highest average rates can be observed in two of the southern regions, and -contrary to mortality rates- no north-south gradient in case fatality rates is present. Correlation analysis showed a significant association between mortality and accident rates at regional level (Spearman's rho = 0.457; p = 0.043), while no association was found between mortality rates and case fatality rates (Spearman's rho = 0.150; p = 0.527).

The results of the linear regression models performed in order to explore the relation between regional traffic mortality rates and socio-demographic variables show that the employment rate (β = 0.759; *p *< 0.001) is the only factor significantly associated with traffic mortality rates within the whole country of Italy (R^2 ^of the model: 0.576). This association is confirmed in both Northern and southern regions It is interesting to note that contrary to the effect of the employment rate, the models indicate different patterns by region as far as concerns the degree of urbanization (beta is positive for northern and negative for southern regions) and per capita income (beta is negative for northern and positive for southern regions). In the multivariate approach performed using the case-fatality index as the dependent variable, the only factor significantly associated (R^2 ^of the model: 0.247) with case fatality rates within the whole country of Italy is the degree of urbanization (β = -0.497; *p *= 0.026). This negative association between urbanization and case fatality is confirmed in the separate models for Northern and southern regions, but the models indicate different patterns in northern and southern regions, as far as concerns the employment rate and per capita income (betas are positive for northern and negative for southern regions).

In Italy as a whole, the traffic accident rate is significantly and directly associated with both the degree of urbanization (β = 0.268; *p *= 0.046) and employment rate (β = 0.789; *p *< 0.001) (R^2 ^of the model: 0.740) (Table 1). The effect of urbanization is much stronger and significant only in northern regions, while a strong and significant positive effect of the employment rate can only be seen in the southern regions (β = 0.826).

**Table 1:** Multiple regressions of traffic mortality rate and case-fatality rate on socio-demographic variables

Dependent variables			Covariates			R^2 ^of the Model
				
			Degree of urbanization	Employment rate	Per capita income	
Traffic mortality rate	*Italy*	Beta	-0.224	**0.759**	-0.064	0.576
		*p*	0.152	**< 0.001**	0.750	
	*Northern Regions*	Beta	0.017	**0.638**	-0.188	0.407
		*p*	1.0	**0.026**	0.479	
	*Southern Regions*	Beta	-0.356	**0.713**	0.052	0.508
		*p*	0.305	**0.047**	0.923	

Case-fatality rate	*Italy*	Beta	**-0.497**	-0.233	-0.039	0.247
		*p*	**0.026**	0.270	0.888	
	*Northern Regions*	Beta	**-0.976**	0.093	0.550	0.582
		*p*	**0.006**	0.715	0.078	
	*Southern Regions*	Beta	**-0.873**	-0.033	-0.167	0.763
		*p*	**0.005**	0.933	0.506	

Traffic accident rate	*Italy*	Beta	**0.268**	**0.789**	0.047	0.740
		*p*	**0.046**	**< 0.001**	0.780	
	*Northern Regions*	Beta	**0.598**	0.032	0.039	0.358
		*p*	**0.040**	0.918	0.898	
	*Southern Regions*	Beta	-0.038	**0.826**	0.309	0.683
		*p*	0.913	**0.011**	0.493	

In the multiple regressions of traffic mortality rates on variables related to traffic, road behaviour, infrastructural characteristics and health care, the factor significantly associated to traffic mortality rate at national level (R^2 ^of the model: 0.362) is the prevalence of alcohol drinking (β = 0.601; *p *= 0.005). Considering separate models for northern and southern regions, we found different patterns for length of highways (betas are positive for northern and negative for southern regions). Anyway, the R^2 ^of the model shows that the model itself does not fill the data very well for northern regions.

In the multiple linear regression models using the case-fatality index as outcome (R^2 ^of the model: 0.157), the only variable significantly associated with case-fatality at national level was the number of circulating vehicles (β = -0.397; *p *= 0.047). In the separate models for northern and southern regions we found different patterns for number of vehicles and length of highways (Table 2). Interestingly, the best model performance (R^2 ^= 0.944) was found in the analysis regarding southern regions indicating an inverse relationship between the case-fatality rate and the length of highways (β = -1.034; *p *= 0.029) and availability of MR-scan (β = -0.541; *p *= 0.045), and a direct relationship with number of vehicles (β = 1.209; *p *= 0.045) and with prevalence of alcohol drinking (β = 1.157; *p *= 0.015).

**Table 2:** Multiple regressions of traffic mortality rate and case-fatality rate on variables related to traffic, road behaviour, infrastructural characteristics and medical care

Dependent Variables			Covariates				R^2 ^of the Model
			Number of vehicles	Length of highways	Availability of MR-scan	Prevalence of alcohol drinking	

Traffic mortality rate	*Italy*	Beta	0.028	0.008	-0.009	**0.601**	0.362
		*p*	0.886	0.982	0.965	**0.005**	
	*Northern Regions*	Beta	-0.044	0.216	-0.18	0.043	0.034
		*p*	0.95	0.725	0.735	0.917	
	*Southern Regions*	Beta	-0.221	-0.421	-0.088	0.662	0.439
		*p*	0.801	0.281	0.856	0.074	

Case-fatality rate	*Italy*	Beta	**-0.397**	-0.192	-0.187	-0.222	0.157
		*p*	**0.047**	0.609	0.405	0.322	
	*Northern Regions*	Beta	-0.403	0.247	-0.141	0.076	0.163
		*p*	0.194	0.615	0.305	0.838	
	*Southern Regions*	Beta	**1.209**	**-1.034**	**-0.541**	**1.157**	0.944
		*p*	**0.045**	**0.029**	**0.045**	**0.015**	

Traffic accident rate	*Italy*	Beta	**0.303**	0.075	0.148	**0.768**	0.631
		*p*	**0.05**	0.768	0.333	**< 0.001**	
	*Northern Regions*	Beta	0.377	-0.101	0.221	0.256	0.142
		*p*	0.227	0.853	0.617	0.438	
	*Southern Regions*	Beta	-0.221	0.562	0.659	-0.599	0.434
		*p*	0.58	0.405	0.076	0.348	

In Italy as a whole, the traffic accident rate is significantly associated with the number of circulating vehicles (β = 0.303; *p *= 0.050) and the prevalence of alcohol drinking (β = 0.768; *p *< 0.001) (R^2 ^of the model: 0.631). In the models with the accident rate as dependent variable, we again observed that the beta coefficients, although not statistically significant, show opposite results for northern and southern regions, with the exception of the availability of MR-scan (positive effect on accident rates in both northern and southern regions).

## Discussion

In Italy, according to our analysis, for the time period 1999–2002, regional differences in traffic mortality, case-fatality and accident rates can be observed. This is consistent with the few studies published in the scientific literature concerning regional differences in traffic accident mortality [6,7].

Regarding the models involving socio-demographic variables, regional differences in the average mortality rate for the years 1999–2002 in Italy appear to be explained by the amount of employed people, while case fatality is associated with the degree or urbanization, and regional variation in accident rates is explained by both the employment rate and the degree or urbanization. In broad terms we found the higher the employment rate, the higher the traffic mortality rate; the higher the degree of urbanization, the lower the case-fatality index; and the higher the employment rate and the degree of urbanization, the higher the traffic accident rates. Interestingly, we found rather different patterns and associations for northern and southern regions.

Baker et al. [8] found in USA that mortality was highest in counties of low population density and was also inversely correlated with per capita income; moreover, Van Beeck et al [6] found in the Netherlands that a higher income level is associated with lower mortality levels. Yang et al. [24] found a linear relationship between decreasing urbanization and increasing SMRs for motor vehicle crash mortality. Our results are not in full agreement with these findings, since we did not find a significant effect of income and urbanization on regional mortality levels. As far as concerns the income, it could be that this variable is responsible for within region variation in accident mortality and morbidity, but data at the provincial level were not available. Instead we demonstrated a strong direct effect of the employment rate (an omitted variable in previous studies) on traffic mortality and accident rates.

Van Beeck et al. [25] and Suriyawongpaisal et al. [5] reported that in developed and developing countries respectively trends of traffic injuries seem to follow trends of economic growth. Our results seem to indicate that economic developments, reflected by employment rates, also have strong effects on traffic accident and mortality rates in Italy, both in northern and southern regions.

Regarding the models involving variables related to traffic, road behaviour, infrastructural characteristics and health care, we found that both traffic mortality and accident rates are influenced directly by the prevalence of alcohol drinking, while the number of circulating vehicles is a strong predictor of case fatality (inverse association) and accident rates (direct association). Again, we found different patterns for Northern and southern regions, especially for the variables, availability of MR-scan and prevalence of alcohol drinking.

Van Beeck et al. [6] reported that in the Netherlands, traffic density and the availability of advanced trauma care (neurosurgery and computerized tomography (CT-Scan)) in the region are the most important predictors (being inversely associated) of regional mortality differences. In our study we used the number of circulating vehicles as a measure of traffic density, and we found similar results as in the Netherlands. On the other hand, we used the availability of MR-scan as a measure of availability of advanced trauma care, and -contrary to the Netherlands- could not show an effect of regional differences in medical care. The Dutch study, however, did not include road behaviour variables, whereas the present study from Italy could analyze the influence of alcohol drinking in a multivariate approach. Our study showed that these road behaviour variables seem to belong to the strongest predictors of regional differences in road traffic accident mortality, and analyses without adjustment for these variables (such as the Dutch study) should therefore carefully interpreted.

It must be considered, however, that the present study has some limitations as well. An important possible source of bias is the so-called omitted-variable problem, which arises when a study does not account for all explanatory variables [6]. In other words, our analysis is based on the availability of variables that could explain, totally or partially, the variability in the outcome variables we were interested in. However, although being more complete than previous studies, some interesting variables are still missing, such as travel speed and velocity at impact of accident, because they are not available at the regional level. These variables might be important explanatory variables whose effects can be seen through the differences in case fatality. As a measure of exposure to alcohol in injured drivers, we used data on prevalence of alcohol drinking, but we were not able to report injured drivers' use of illicit drugs, because this is not routinely collected after a traffic accident in Italy.

Another potential limitation of this study is the accuracy of the data; even though we used data sources from established national institutes (e.g. the National Institute of Statistics and the National Institute of Health), one can argue that for some of them possible errors could occur. As an example, the employment rates in the southern regions could have underreported the real employment levels in those regions.

The period considered in our study is 1999–2002, and we collected data on possible determinants of traffic accidents and mortality on the basis of availability of potential explanatory variables (average per capita income calculated on the period 1995–2001; availability of highways in the period 2001–2002). On our opinion, this time-differences in data periods did not affect the results of our analysis, due to the fact that we considered mean values of these variables, in order to avoid possible measurement bias. In all studies based on existing data sources, the reported findings must be interpreted with care, because of the possibility of registration artefacts. A possible source of bias could be due to regional differences in accident reporting, and this could be an explanation of the deep differences between northern and southern regions, especially for the numbers of injured people. The impact this could have is that regional differences in registration thresholds may exist, i.e. there may be less recording of injuries in the more rural parts of a country. If this is so, it would lead to an artificial increase in case-fatality [6]. Differential underreporting of accidents by region may therefore have affected our analyses on accident and case fatality rates. However, this has probably not affected the mortality rates, because of the standardisation of procedures of recording a death due to traffic accidents at the national level. We therefore argue that this potential registration bias has not exerted large influences on our main results.

As major adding to the literature, we have demonstrated the effect of employment rates and alcohol use on regional differences in both traffic accident mortality and accident rates. Although the results must be interpreted with care and further research should support well-tailored prevention strategies (e.g studies examining the role of more explanatory factors), our study has shown the need for regional policies to improve road behaviour and reduce traffic accident and mortality rates in identified high-risk areas.

## Conclusion

Our study confirms the validity and reliability of using current data for studying the influence of socio-economic determinants on road accident rates, since the quality of this type of data has much improved at the national level.

Moreover, it shows that regional differences in the average mortality rate for the years 1999–2002 in Italy appear to be explained by the amount of employed people, while case fatality is associated with the degree or urbanization, and regional variation in accident rates is explained by both the employment rate and the degree or urbanization.

Finally, traffic mortality and accident rates are influenced directly by the prevalence of alcohol drinking, while the number of circulating vehicles is a strong predictor of case fatality (inverse association) and accident rates (direct association). These results could be used for preventing purposes (primary and secondary prevention) by policy and decision makers.

## Competing interests

The author(s) declare that they have no competing interests.
